# Interactive mirrOring Games wIth sOCial rObot (IOGIOCO): a pilot study on the use of intransitive gestures in a sample of Italian preschool children with autism spectrum disorder

**DOI:** 10.3389/fpsyt.2024.1356331

**Published:** 2024-06-28

**Authors:** Silvia Annunziata, Laura Santos, Arianna Caglio, Alice Geminiani, Elena Brazzoli, Elena Piazza, Ivana Olivieri, Alessandra Pedrocchi, Anna Cavallini

**Affiliations:** ^1^ IRCCS Fondazione Don Carlo Gnocchi, Milan, Italy; ^2^ Department of Electronics, Information and Bioengineering, Politecnico di Milano, Milan, Italy; ^3^ Institute for Systems and Robotics, Instituto Superior Técnico, University of Lisbon, Lisbon, Portugal; ^4^ Centro Benedetta d’Intino Onlus, Milan, Italy

**Keywords:** autism spectrum disorders, social robot, technological rehabilitation, children, communicative gestures

## Abstract

**Background:**

Autism Spectrum Disorder (ASD) is a neurodevelopmental disorder characterized by persistent deficits in social communication, social interaction, and restricted behaviors. The importance of early intervention has been widely demonstrated, and developmental trajectories in ASD emphasize the importance of nonverbal communication, such as intransitive gesture production, as a possible positive prognostic factor for language development. The use of technological tools in the therapy of individuals with ASD has also become increasingly important due to their higher engagement and responsiveness to technological objects, such as robots.

**Materials and methods:**

We developed a training protocol using the humanoid robot NAO, called IOGIOCO (Interactive mirroring Games wIth sOCial rObot), based on the use of intransitive gestures embedded in naturalistic dialogues, stimulating a triadic interaction between child, robot and therapist. The training was divided into six levels; the first 2 levels were called “familiarization levels,” and the other 4 were “training levels”. The technological setup includes different complexity levels, from mirroring tasks to building spontaneous interactions. We tested the protocol on 10 preschool children with ASD (aged 2–6 years) for 14 weeks. We assessed them at recruitment (T0), at the end of training (T1), and after 6 months (T2).

**Results:**

We demonstrated the tolerability of the protocol. We found that one group (n=4, males and 2 females) reached the training level, while another and group (n=6 males) remained at a familiarization level (mirroring), we analyzed the results for the two groups. In the group that reached the training levels, we found promising results, such as an improvement in the Social Adaptive Domain of the ABAS-II questionnaire between T0 and T2.

**Conclusion:**

While current results will need a Randomized Controlled Trial to be confirmed, the present work sets an important milestone in using social robots for ASD treatment, aimed at impacting social and communication skills in everyday life.

## 1 Introduction

Autism Spectrum Disorder (ASD) is a neurodevelopmental disorder. It is characterized by impairment in everyday life due to two main core deficits or symptoms: the first cluster (criterion A) is characterized by the presence of persistent deficits in social communication and social interaction, which comprises deficit in verbal (i.e., language delay or stereotyped language) and non-verbal communication (abnormal or diminished eye-contact, hypomodulation of facial mimicry, lack or limited use of gestures), difficulty in initiating, maintaining and ending social interaction. The second cluster of symptoms (criterion B) is characterized by the presence of restricted, stereotyped, and repetitive patterns of behaviors, including pervasive interests that compromise sociality, unusual sensory interests or patterns of hypo and/or hyperreactivity to sensory stimuli, and/or the presence of stereotyped movements (1, 2).

It is a disorder on the rise, affecting approximately 1 in every 36 8-year-old children in the U.S (3)., and its prevalence has been steadily increasing over the last decades. In Italy, the disorder is estimated to be present in one in 77 children among 7- to 9-year-olds (4).

Despite the ability to reliably diagnose ASD in children as young as 24 months of age, the diagnosis remains delayed in many children. According to Daniels and colleagues (5), to solve this problem, it is crucial to increase awareness about the disorder, enhance routine screening, and improve clinical practice. In the last decade, this has been a great effort of the researchers in this field and has led to significant results in terms of the identification of screening tools, such as M-CHAT, First Years Inventory (FYI), and Quantitative- Checklist for Autism in Toddlers (Q-CHAT) (6) and application of new screening protocols. In Italy, this aspect has also been extensively studied, with a particular focus on the development and research of primary and secondary screening methods for the identification of early risk cases, which can then be referred for specialist diagnostic assessment in the first two or three years of life (7, 8).

The first sign that often warns parents is expressive language delay: the estimated prevalence of language delays/disorders is 87% in 3-year-old children with ASD (9). However, many early signs besides language delay can be identified, such as the lack of socio-communicative initiative and response to social bids. The impairment in communication early in life is related not primarily to speech delay but to a deficit of non-verbal communication (e.g., eye contact or gestures. Previous research showed that English-speaking children with ASD have a delay in the development of gestures compared to age-matched peers with typical development and developmental delay (10, 11). This delay affects particularly “proto-declarative” gestures such as “pointing” (12). ASD children tend to use less cultural-related gestures for communication purposes (such as raising their thumbs to hitchhike) (10, 11) as well as iconic gestures (10). Ham and collaborators (13) hypothesized that individuals with ASD may have a selective delay in using intransitive gestures for communicative purposes (such as, for example, waving a hand to greet). Moreover, they recognize transitive gestures better than intransitive ones (14).

In addition, it has been suggested that ASD children could have some impairments in observational learning and imitation that may result in poor observation skills (15) and more difficulty in understanding and imitating gestures. This hypothesis aligns with previous research, which has shown that people with ASD imitate gestures that have a purpose more successfully than gestures without communicative or descriptive meaning (16).

An early diagnosis is fundamental to allow the activation of an early intervention program. Acting early in child development is nowadays considered a priority, as it has been demonstrated that the timing of intervention is crucial in predicting the response to treatment (17, 18). In recent decades, many early intervention models have been developed. A recent meta-analysis (19) showed that most of these models have been proven effective in addressing core symptoms of ASD. However, more research on this topic is needed (20).

In literature, data on rehabilitation trainings that promote the use of intransitive gestures in ASD are scarce. However, it is known that early intervention in gesture learning can have positive effects on children’s verbal and non-verbal communication and social skills. Better imitation skills in children with ASD are often associated with verbal language development in the short and long term (21). Therefore, these results suggest that imitation skills and gesture production may play a fundamental role in these children’s communication and language development (22).

In recent times, alongside implementing behavioral and developmental interventions conducted in a naturalistic setting, there has been a concurrent increase in the study of alternative technological approaches (23). The rationale of these approaches centers on exploring children’s interest in technological devices to foster their involvement and motivation toward social interaction and participation (24, 25). People with ASD tend to have great difficulty paying attention to the variability and multiplicity of signals that characterize human social interaction (e.g., mimicry, gestures…), an aspect that can lead to a reduced interest in interacting with a person. However, they demonstrate active engagement, attention, and reactivity towards technological objects (24–26). Moreover, humanoid robots are stylized, “toy-like,” non-invasive, and characterized by a simple use set-up (27).

In this way, robotic applications have been developed for patients with motor, social, and cognitive disabilities to promote their participation and involvement during therapy (25, 28). A significant part of the literature regarding robotic use in ASD participants is related to intervention. The principal types of interventions focus on joint attention (29–32), sensory processing (33), imitation training (34), and emotion recognition (28, 35–40). Other works focused on a global improvement of social skills (41–45). In Italy, studies using humanoid robots in ASD intervention are focused mainly on emotion recognition (37, 40), motor skills (46) and even on the development of robotic assessment tools (47).

Few studies developed an intervention focused on gesture recognition and production in ASD. So and colleagues (48, 49) developed an intervention protocol based on intransitive gesture recognition and imitation in a robot-child intervention. They found out that during a robot-based intervention, ASD children produced more accurate and/or appropriate intransitive gestures than those in the waitlist control group (49). This finding highlights two possible implications: on one side, it could be postulated that in such a training, the humanoid robot can replace a human therapist; on the other hand, these findings underline that there is no actual superiority of robot intervention over human intervention in gestural learning as participants’ gestures repertoire increased in both human and robot intervention (50). This aspect is controversial, as many studies, as reported in the review of Saleh et al. (51), demonstrated the positive effect of robotic intervention in ASD on social skills. From this perspective, the use of a social robot continues to be an exciting area of research in the rehabilitation of children with ASD.

Studies focused on gesture training are mainly oriented on gesture teaching in a child-robot interaction: the assumption in this approach is that gesture training only with a social robot can promote gesture recognition in a therapy setting and its generalization in everyday life and human-to-human interaction (48). Otherwise, generalization is one of the most critical aspects of ASD intervention, mainly when a behavioral approach is used (52). As there is growing awareness about the importance of a more naturalistic approach in ASD intervention, we decided to exploit the motivation given by technological tools to create a triadic interaction, where the robot, the child, and an early interventionist interact. To the best of our knowledge, a few studies have shown the use of humanoid robots in triadic therapies with ASD children (53–55), and none of them have focused on gesture training.

Considering all these, within the broader national context, more research is still needed to explore the potential of robotic approaches to gesture training to enhance social interaction in preschool age. This study aims to address this gap in the existing body of knowledge. We, therefore, designed and implemented a protocol on intransitive gesture training (named “Interactive mirroring Games wIth sOCial rObot – IOGIOCO”) for preschoolers that uses a triadic relationship between a child, a humanoid robot called NAO, and a therapist to foster learning and generalization of communication skills. In our approach, robotic intervention is used in synergy with human intervention, not substituting it.

Given our primary goal, we established two research questions for our pilot study:

- RQ1: Is the IOGIOCO protocol feasible and acceptable in a sample of ASD preschoolers?
- RQ2: Given the preliminary results, what should be the main directions for an improved intervention protocol and a more robust trial?

## 2 Materials and methods

### 2.1 Participants

We included subjects with a diagnosis of Autism Spectrum Disorder made according to DSM-5 criteria and confirmed by Autism Diagnostic Interview-Revised, ADI-R (56) and the Autism Diagnostic Observation Schedule – II edition, ADOS-2 (57), aged under 72 months at the time of recruitment, that were already in treatment with psychomotor and/or speech therapy in the Child Neuropsychiatric and Rehabilitation Unit of Don Gnocchi Foundation “Santa Maria Nascente” in Milan (Italy).

We excluded patients with preterm birth, pregnancy complications or perinatal injury history, major facial peculiar characteristics, malformations or neuroradiologic alterations, epileptic syndromes, known congenital infections, metabolic or genetic diseases. Developmental skills were assessed using the Griffiths-III Scale (58). Developmental delay was defined by a General Quotient (GQ) lower than 70.

We enrolled 14 children from February 2020 to September 2021. Due to the COVID-19 pandemic, the study stopped in March 2020 and re-started in November 2020. During the experimental phase, three children dropped out due to family organizational difficulties, 11 completed the training period, and one did not undergo the T2 post-test.

The final sample includes ten subjects, eight males and two females; a synthesis of the characteristics of the sample is shown in **Table 1**. All patients except one have a psychomotor delay (GQ at Griffiths Total Scale < 70).

**Table 1:** Characteristics of the sample.

	I.D.	Gender	Age (months)	Griffiths GQ	ADI SI	ADI CL	ADI RRB	ADOS mod	ADOS SA	ADOS RRB	ADOS CS
**Training**	09	M	62	65	1,53	1,39	0,83	1	1,1	1,5	7
	08	M	67	58	1,4	1,5	1,3	2	1,8	1,25	9
	06	F	50	**75**	1,4	1,5	1,3	2	0,8	1	6
	05	F	63	55	1,85	1,86	0,5	1	2	1,25	9
**Familiarization**	01	M	55	46	1	0,8	1,5	1	1,6	1,5	7
	04	M	68	37	1,4	1,86	1	1	1	0,75	6
	07	M	55	32	1,87	1,7	0,8	1	1,1	1,75	6
	02	M	56	37	1,4	1,75	1,5	1	1	1,5	6
	03	M	63	41	1,54	0,6	0,67	1	1,5	0,75	6
	11	M	50	42	1,5	1,75	0,5	1	1	1	5
**Mean**		80% (males)	58	48,8	1,5	1,5	1		1,3	1,2	6,7
**S.D.**			7,2	14	0,3	0,4	0,4		0,4	0,3	1,3
**Range**			50–68	32–75	1–1,87	0,6–1,86	0,5–1,3		0,8–2	0,75–1,75	5–10

ADI-R SI, Social interaction; CL, Communication and language; RRB, Restricted and repetitive behaviors; mod, ADOS module; SA, ADOS Social Affect; RRB, ADOS Restrictive Repetitive Behaviors; CS, ADOS Comparative Score; GQ, Griffiths-III General Quotient; SD, Standard Deviation.

We did not have a control group in the study, as it is a pilot study. However, we retrospectively divided the sample into two subgroups according to the level reached during the training period, as explained in paragraph 2.3.2.

### 2.2 Technological set-up

The study was conducted in a small therapy room in the Child Neuropsychiatric and Rehabilitation Unit of the Don Gnocchi Foundation in Milan. The set-up included a social robot, a computer, and a camera for movement tracking. The three components were used to implement different exercises in a triadic interaction between the adult, the child, and the robot.

NAO (Aldebaran Robotics Company) was the social robot chosen. It is a humanoid-anthropomorphic robot 50-cm-tall. It has 25 degrees of freedom on the whole body and sensors (touch sensors, microphones, and two cameras). It also has 16-eye LEDs and two loudspeakers, which are helpful for multi-sensory interaction. The camera used was a Kinect 2.0 depth camera (Microsoft – Redmond, Washington, USA), which could detect people and identify the 3D positions of their joints. The Kinect identifies 25 joints whose ensemble is called skeleton.

**Figure 1:**
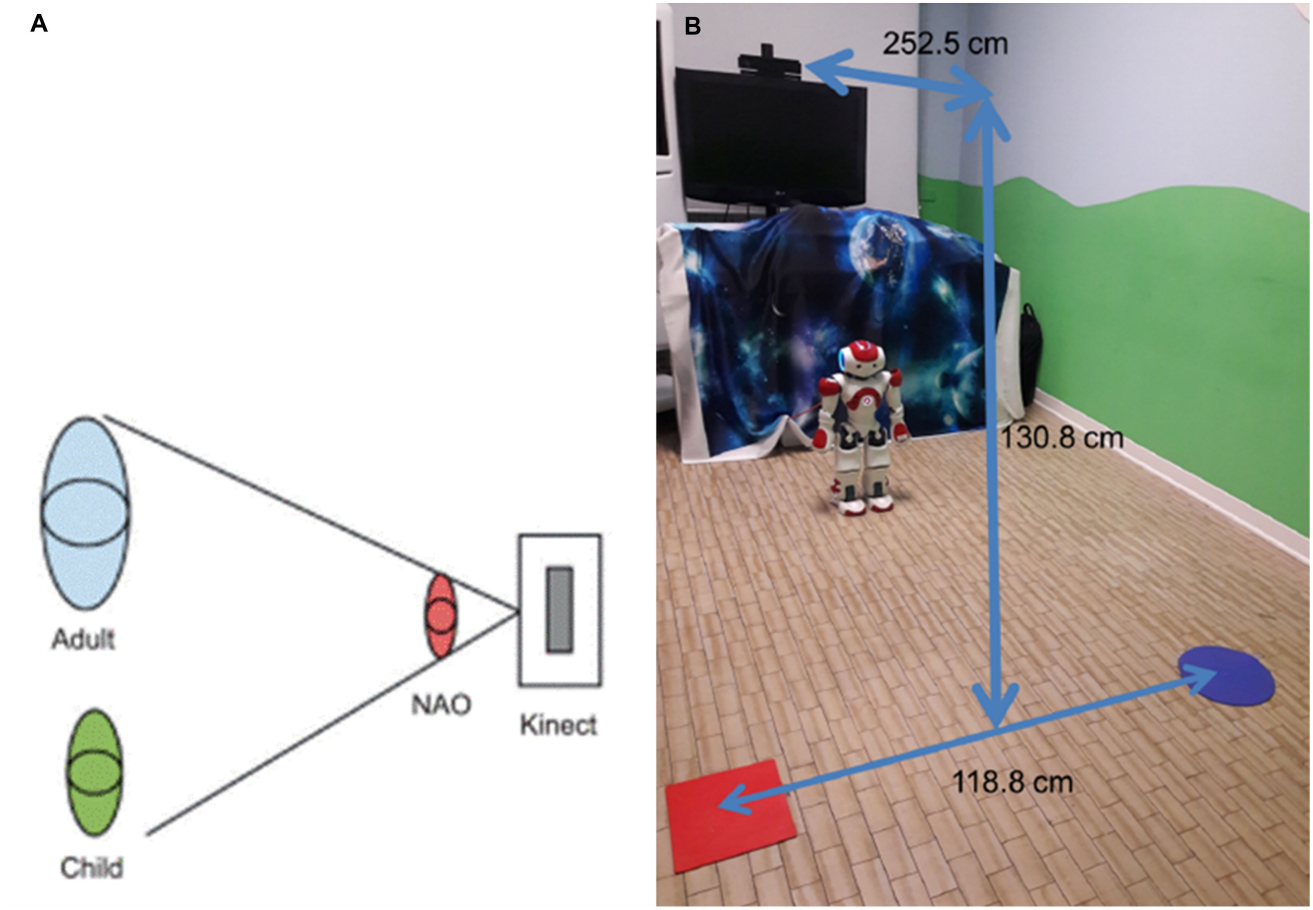
Positions of the participants (adult-child-robot) and of the motion tracking system (Kinect) **(A)** and image of the clinical setting **(B)**. Image of the participants’ positions as reported in (59), reproduced with the authors’ permission. Image of the room reproduced with permission from IRCCS Fondazione Don Gnocchi of Milan (Italy).

During the sessions, the three actors were positioned in a triangle, with the Kinect camera positioned above the NAO robot and in front of the two people so that the Kinect could identify both subjects (**Figure 1**). To differentiate the child from the therapist, the therapist wore a red T-shirt, and she was identified through masking techniques from computer vision algorithms; the child (the person without the red T-shirt) was automatically identified as the other person in the room. A TV screen was positioned behind the robot, and a sheet with an image of the space was placed in the background.

**Figure 2:**
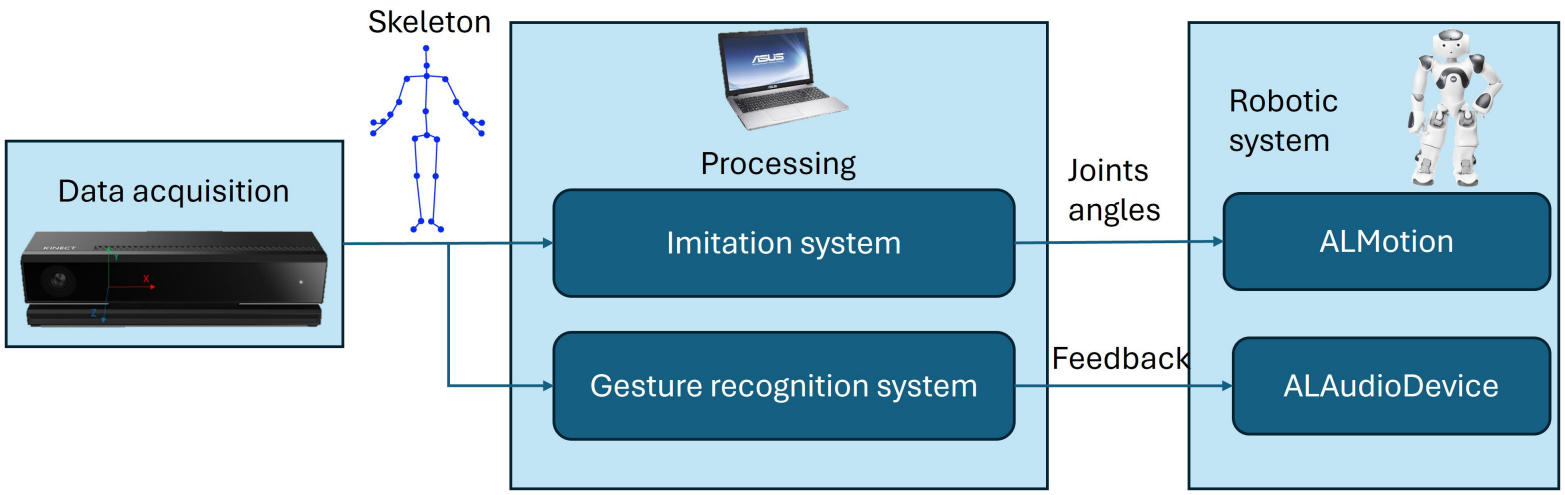
Block diagram of the entire system. The Kinect camera acquires the data. This data is constituted by the 3D joint positions’ of each detected person, and it is called skeleton. The skeleton is passed both to the Imitation System and the Gesture Recognition System, which are responsible for real-time mirroring and identification of the gesture mirrored, respectively. Through the ALMotion and ALAudioDevice modules, the two systems pass the joint angles and the feedback to the robot.

Two systems worked in parallel in the background: an imitation system and a gesture recognition system (**Figure 2**). They were activated according to the exercises chosen by the therapist. The imitation system allows the robot to perform real-time mirroring. First, the Kinect identified the skeleton of one of the participants (the one to be mirrored). Based on geometry, the trajectory of angles between the upper limb segments (arm-forearm, arm-trunk) were extracted from joint trajectories. Then, the angle values were used as control variables for the robot NAO through the module *ALMotion* so that he could reproduce the movement. A full explanation of the mirroring system is present in our previous works (59, 60).

Regarding the gesture recognition system, for each mirrored person, the beginning and end of the gesture were identified through Kinetic Energy considerations, applied to the skeletons recoiled with the Kinect. After defining the two time points, the skeletons acquired were concatenated to construct an image. This image passed through a Neural Network (ResNet) that identified the gesture performed. If the gesture performed corresponded to the therapist’s selected gesture, feedback was given to the robot through the *ALAudioDevice* module. Further details on the gesture recognition system are provided in (61).

#### 2.2.1 INTERFACE

The therapist controlled the entire system through a Graphical User Interface (GUI) running on a Personal Computer. Alternatively, a tablet was also used. The GUI has been structured to be simple, straightforward, and immediate to make the session as fluid as possible. The therapist could write his/her name, the child’s name, and the session number in the first panel and then access a tab to select the various levels of the protocol. Once the level is chosen, the various specific commands can be sent to NAO (for example, activation of LEDs, specific movement, and gestures).

### 2.3 Therapy protocol (IoGioCO)

#### 2.3.1 Therapy levels

**Figure 3:**
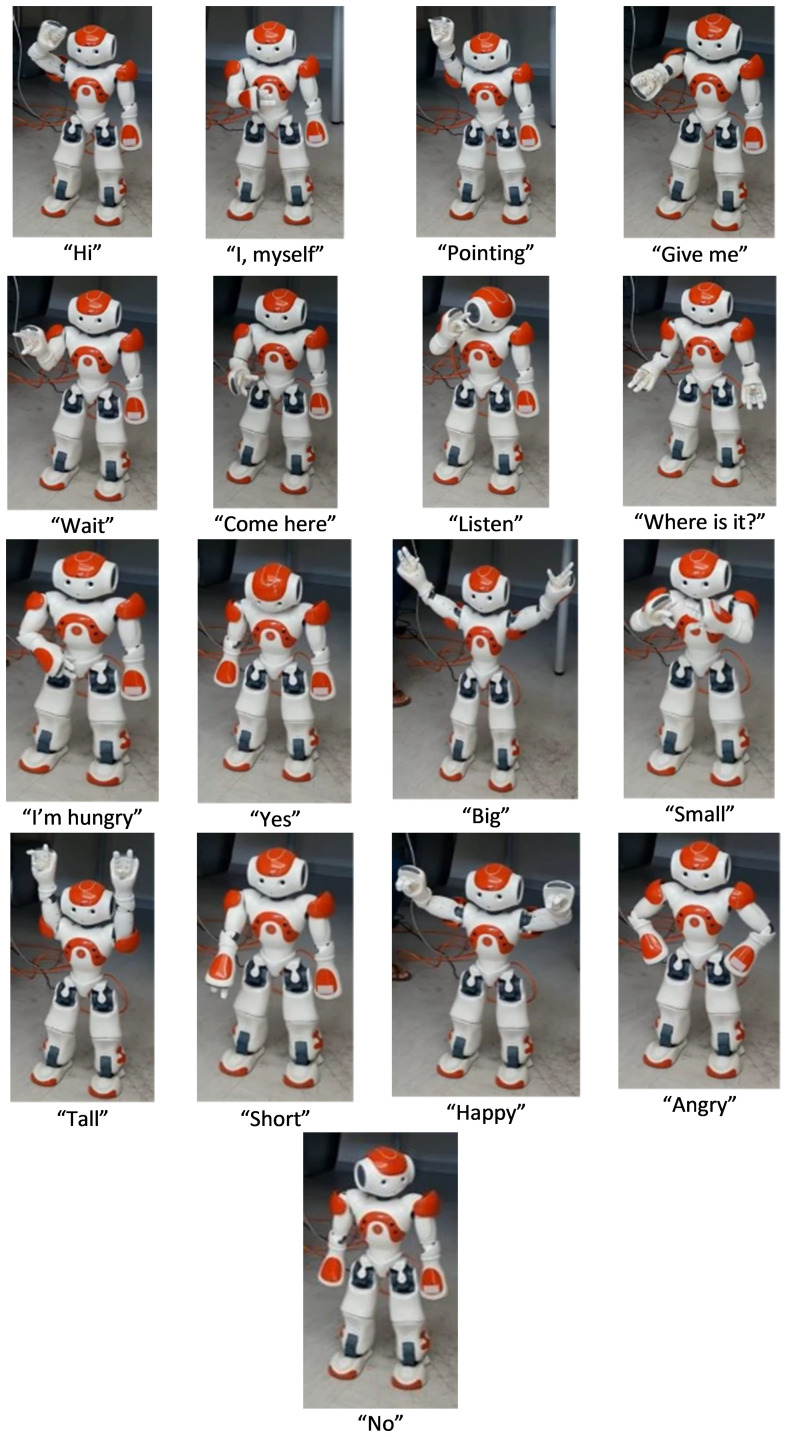
Pictures of NAO doing the selected gestures. Images of the robot reproduced with permission from IRCCS Fondazione Don Gnocchi of Milan (Italy) and Politecnico of Milan (Italy).

The training protocol consists of five phases, with a final level. The first two levels aim to get familiar with NAO and increase compliance, named “familiarization levels” from now on. Levels 3, 4, and 5 contain training sessions, including new gesture teaching and progressive generalization of the gestures taught within short narratives and different daily-life scenarios. These levels are referred to as “training levels” in the rest of the manuscript. In these levels, we included 17 intransitive communicative gestures used in everyday life: *Hi/Myself/Pointing/To Give/Wait/Come here/Listen/Where is it?/Hungry/Yes/No/Big/Little/Tall-High/Short/Happy*/*Angry* (**Figure 3**). The details of each level are presented next.

**Level 1– Familiarization**. As some children can be worried or frightened by the new unpredictable situation, this phase has been introduced to facilitate a smooth and gradual introduction to the robot and the setting, adapting the situation and the stimuli provided according to the child’s comfort and willingness to engage. The therapist controls the robot, and NAO can illuminate and talk, pronouncing pre-set or real-time personalized sentences entered via the therapist’s GUI. Additionally, NAO can present the communicative gestures in the protocol without any specific request to the child.

**Level 2 – Mirroring.** NAO can recognize and imitate the child’s movements so that the child can experience the mechanism of real-time mirroring. The experience is structured without any specific request.

**Figure 4:**
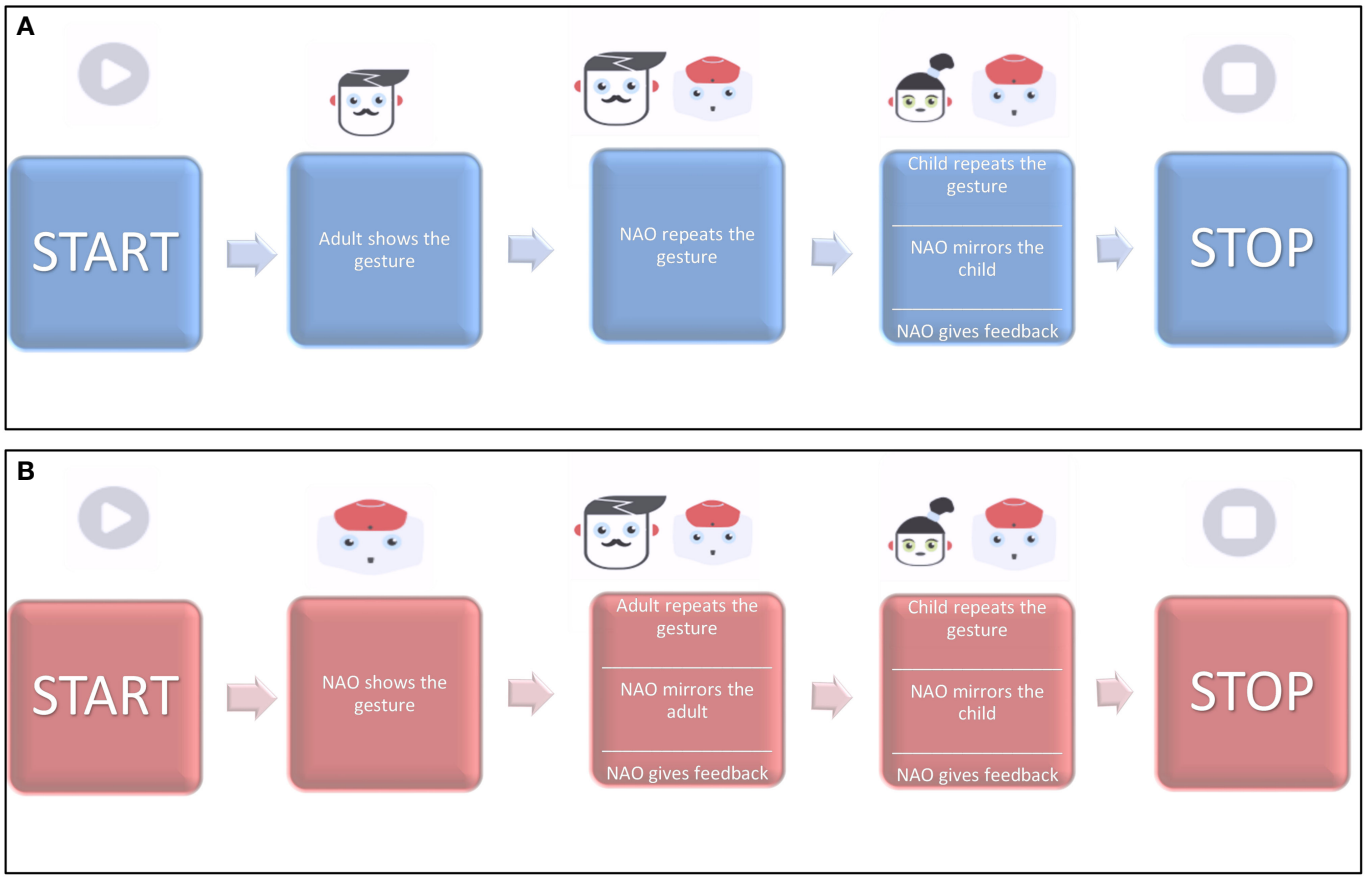
Block diagrams of the training modes for Levels 3–4. **(A)** Adult-Coach protocol and **(B)** Robot-Coach protocol. Image adapted from (59), with the permission of the authors.

**Level 3 – Gesture Training**. The child is asked to repeat the 17 communicative gestures included in the protocol. The therapist decides which gestures to work on with the child in each session and through which specific modality, based on who controls the movement execution: Therapist Coach (TC) or Robot Coach (RC) (see **Figure 4**).

In RC mode, the gesture is introduced by the robot and repeated first by the therapist and then by the child. Here, NAO performs a real-time mirroring of the gestures and gives positive (“Fantastic!” or “Wonderful!”) or negative feedback (“Let us try again!”).In TC mode, the roles are inverted: the therapist shows the gesture first, and then the robot and the child are asked to repeat it.

**Figure 5:**
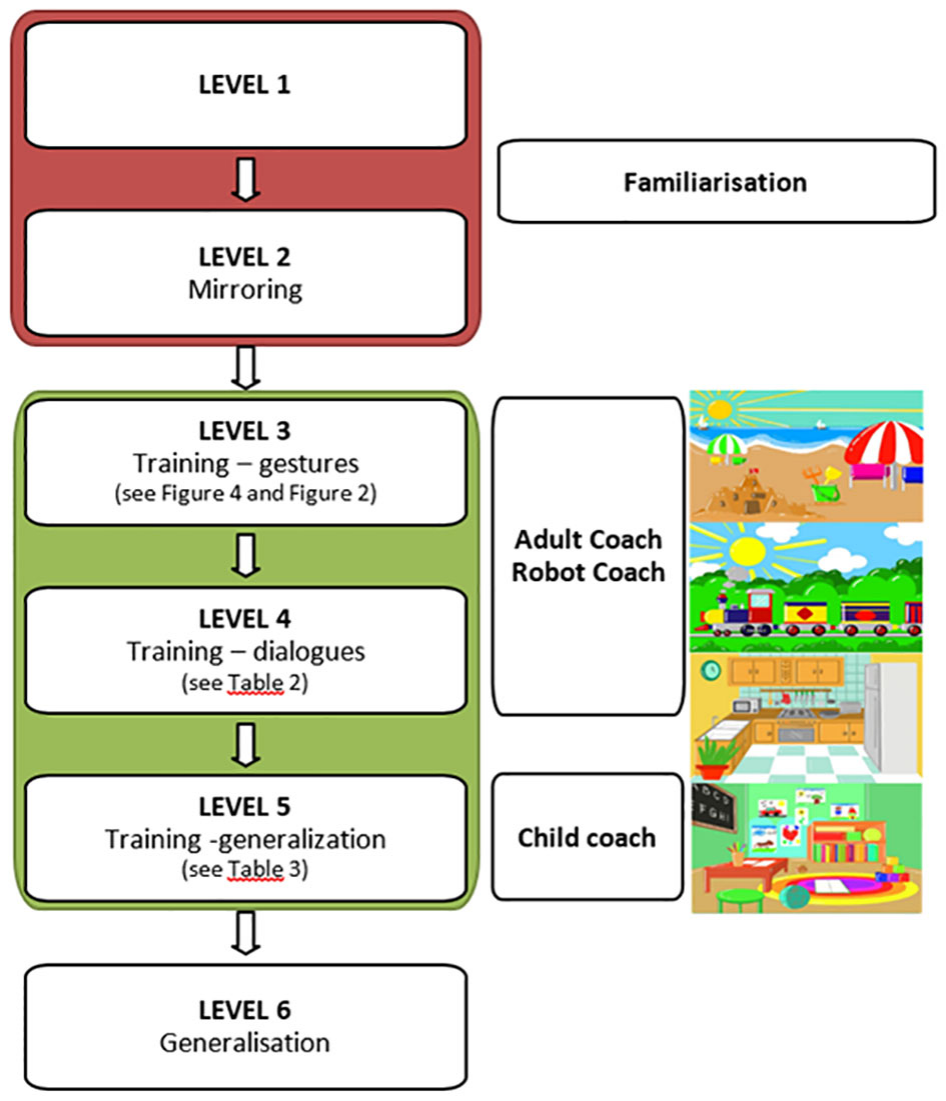
Clinical protocol design.

**Level 4– Training (Dialogues)** The child is asked to repeat the communicative gestures selected through the TC or RC modes and structured in short pre-set dialogues (see **Table 2**). Each dialogue is set in different scenes from everyday life, such as the kitchen, the bedroom, the school, the sea, and the train. When selected, their settings are presented on a screen behind the robot to help the child contextualize (see **Figure 5**).

**Table 2:** Examples of dialogues (Level 4) (English adaptation).

ROBOT COACH	
***NAO***	I am hungry
***ADULT***	I am hungry too, (reproducing the gesture)
***CHILD***	I am hungry, (reproducing the gesture)
***NAO***	COME to the kitchen
***ADULT***	I COME to the kitchen (reproducing the gesture)
***CHILD***	I COME to the kitchen (reproducing the gesture)
THERAPIST COACH	
***ADULT***	GIVE me a dish
***NAO***	I give you a plate (reproducing the gesture)
***CHILD***	I give you a plate (reproducing the gesture)
***ADULT***	WAIT, it is not ready
***NAO***	I WAIT, (reproducing the gesture)
***CHILD***	I WAIT, (reproducing the gesture)

**Level 5– Training (Generalisation)**. This level is based on the child’s initiative. RC and TC modes are not used anymore, and the child is leading the activity. The main goal of this level is to build a meaningful dialogue that is as fluid as possible between the child and the robot based on the learned gestures. There are some pre-set sentences and gestures related to the same scenes presented in level 4 that the therapist can choose to facilitate the interaction (see an example in **Table 3**).

**Table 3:** Examples of sentences (Level 5, kitchen setting) (English adaptation).

**HI**	• **Hi, how are you?** • **HELLO, I am fine**
**POINTING**	• LOOK, we are in the kitchen • Let’s GO to the kitchen • THERE IT IS
**ME**	• I want to eat • I am thirsty • ME too
**GIVE ME**	• Can you GIVE ME something to eat? • Can you GIVE ME something to drink? • I will GIVE you right away
**COME**	• Come on, COME to the table • Come on, COME to the kitchen • I am coming too
**WAIT**	• WAIT, it is not ready yet • Okay, I WAIT
**LISTEN**	• LISTEN, the timer’s ringing, it is ready • I LISTEN too
**WHERE**	• WHERE do we sit? • WHERE do we eat? • Where are we?
**HUNGRY**	• I am hungry, and you? • I am HUNGRY too
**BIG**	• GIVE ME a BIG dish
**SMALL**	• GIVE ME a SMALL dish
**HAPPY**	• I am HAPPY, and I have a full belly
**ANGRY**	• I am ANGRY; I want to eat
**YES**	• YES, I am hungry too • YES is now ready • YES okay
**NO**	• NO, it is not ready yet • NO, I do not like it • NO, I do not want it

**Final Level –** At this level, new daily life scenarios that are different from the previous ones are presented. Here, the training focuses on the child’s spontaneous initiative and generalization skills in using the 17 communicative gestures taught; moreover, this level aims to sustain the triadic relationship and dialogue in a new setting.

#### 2.3.2 Sessions

The intervention lasted 14 weeks for each subject enrolled, with weekly sessions (in the initial protocol, we planned two sessions per week, but due to the reorganization of accesses and spaces due to the COVID-19 pandemic, it was reduced to once per week).

Each session lasted 20–30 minutes, depending on the child’s attention span and engagement with NAO. We decided not to set a predetermined number of sessions for each level, as each child needed to adapt well to a level as a prerequisite to access the following ones.

The transition from one level to the next was based on the child’s compliance with the tasks at the subsequent level. In particular, the child’s interest and confidence in the robot influenced the transition from Level 2 to Level 3, which involved a shift from familiarization to training levels. When the child demonstrated focused attention on the robot and the therapist without signs of fear, the child was ready to progress to the training levels. The transition from Level 3 to the subsequent levels was contingent upon the child’s mastery of the preceding level. In particular, the following criteria were considered:

- Level 3 to level 4: Imitation of most of the proposed gestures (not based on motor performance, but on communication intentionality) and turn-taking in imitation proposed by the therapist and the robot.
- Level 4 to level 5: Initiation of turn-taking in most dialogues proposed, with emerging initiative from the child.

### 2.4 Pre-post training evaluation

Children were evaluated at T0 - baseline, T1- end of the training, T2 - six months after the completion of the training.

At the time of the enrolment, they were assessed using:

- ADOS-2, a semi-structured standardized observation to test the presence and the level of symptoms of Autism Spectrum Disorder (62);
- ADI-R is a semi-structured interview conducted with the caregivers to test the clinical history and the presence of symptoms and behaviors related to Autism Spectrum Disorder (56).

The mean and standard deviation for each ADOS and ADI domain were obtained by dividing the total score by the number of items included to accommodate for differences in the modules and diagnostic algorithms, which are differentiated depending on the child’s developmental and language levels.

Moreover, at T0, T1, and T2, children were assessed using the following primary outcome measures:

- Griffith’s – III Edition is a clinical scale to assess psychomotor development in children under 72 months of chronological age. We considered the General quotient and the Communication Scale.
- ABAS-II is a questionnaire filled by the caregiver to assess adaptive skills in everyday life; it comprises three domains, Social (SAD), Conceptual (CAD), and Practical (PAD) Adaptive Domains, and 11 subdomains.
- MacArthur-Bates Communicative Development Inventories – Words and Gestures (MB-CDI) (Italian adaptation), a questionnaire filled by the caregivers, in which we just considered the part related to gestures (number of gestures, that contains: (A) first communicative gestures, (B) games and routines, (C) actions with objects and imitation of the adult, (D) games of pretending and (F) games of pretending with objects.)

All the examinations were conducted by clinicians (a medical doctor and a psychologist) with expertise in child neuropsychology and ASD diagnosis. They were not blinded to the treatment but were different from the therapist.

The study was performed with written informed consent of the subject’s parents. The study was approved by the Ethics Committee of Fondazione Don Gnocchi IRCCS “Santa Maria Nascente” of Milan (number 6_25/07/2019).

### 2.5 Statistical analysis

We aimed to identify any significant improvement of the measured variables between the pre-(T0), post-(T1) test, and post-follow (T2) up test in the whole group. Given the small sample size, we used non-parametric tests for the statistical analysis, such as the Friedman Test, to see if there were significant differences longitudinally between pre-, post-, and follow-up phases for the seven analyzed variables (Griffiths B: scale and GQ; McArthur: number of gestures; ABASII: DAC, DAS, DAP, GAC). Furthermore, in the cases in which a significant difference was found, we applied the Wilcoxon test to understand in which phase the difference was significant.

As not all the children reached the training levels (levels 3–5), we decided to further divide the group into two subgroups: a *training group* (4 children that reached levels 3–5) and a *familiarization group* (6 children that stayed at levels 1–2). This division was just done for analysis purposes, not being pre-established in the study design. For the analysis of these two groups, we continued to apply non-parametric tests; however, since the sample size is very small (less than five subjects in the training group), the conclusions will have a limited statistical meaning.

To understand better the differences between the *training* and the *familiarization group*, we performed a Mann-Whitney U Test for each variable acquired at T0. Finally, we have done a Friedman Test, followed by a Wilcoxon Test in case of significant results, in each subgroup (training and familiarization group) to understand if there was a difference in the developmental trajectories. All statistical tests were done through the MATLAB software.

## 3 Results

Ten children constituted the total sample of our study: fourteen were recruited, three dropped out due to familiar difficulties raised after the pandemic, and one did not attend the T2 post-test and was not included in the final sample. There were no dropouts due to refusal to attend sessions. However, only some of the patients reached the training levels.

Four recruited subjects reached the training levels (three reached level 5, and one reached level 3), while the other six remained at familiarization levels (levels 1 or 2). A summary of the results for each subject is available in **Supplementary Table 1**.

Considering the whole group of 10 subjects, we found no statistical differences in the pre and post-training evaluation. Moreover, we found a trend of improvement in Griffiths (Scale B and General Quotient) and ABAS II (Cognitive, CAD and Practical, PAD, Domains) that does not reach statistical significance for p<0.05 when using a Friedman Test.

As only some of the subjects underwent the training levels, we, therefore, decided to divide the results’ analysis into two groups, according to the access at the training phase: a *training* and the *familiarization* group. The *training* group comprises 4 participants, two males and two females, while the *familiarization* group comprises six males.

Through the Mann-Whitney U Test, we verified that at T0, all the variables evaluated longitudinally, except the number of gestures, were significantly different (p<0.05) between the familiarization and the training group. Interestingly, the children that reached the training level differed from the familiarization group for developmental and adaptive skills (ABAS II and Griffiths) but not in terms of age and level of autistic symptoms as measured by diagnostic measures (ADI-R and ADOS-2) (**Table 4**).

**Table 4:** Clinical differences at baseline (T0) between the training and familiarization groups.

	p - values
**Griffiths - B scale**	0.010*
**Griffiths - GQ**	0.038*
**McArthur - N° gestures**	0.067
**ABAS II – GAC**	0.029*
**ABAS II – CAD**	0.010*
**ABAS II – SAD**	0.022*
**ABAS II – PAD**	0.038*
**ADI SI**	0.933
**ADI CL**	0.971
**ADI RRB**	0.267
**ADOS SA**	0.533
**ADOS RRB**	1.000
**ADOC CS**	0.105
**Age at recruitment (T0)**	0.648

The asterisks indicate significant difference (*p ≤ 0.05).

ADI-R Social interaction (SI); Communication and language (CL); Restricted and repetitive behaviors (RRB), ADOS module (mod); ADOS Social Affect (SA); ADOS Restrictive Repetitive Behaviors (RRB) ADOS Comparative Score (CS); Griffiths-III General Quotient (GQ); General Adaptive Composite Score (GAC), Conceptual Adaptive Domain (CAD); Social Adaptive Domain (SAD); Practical Adaptive Domain (PAD).

**Figure 6:**
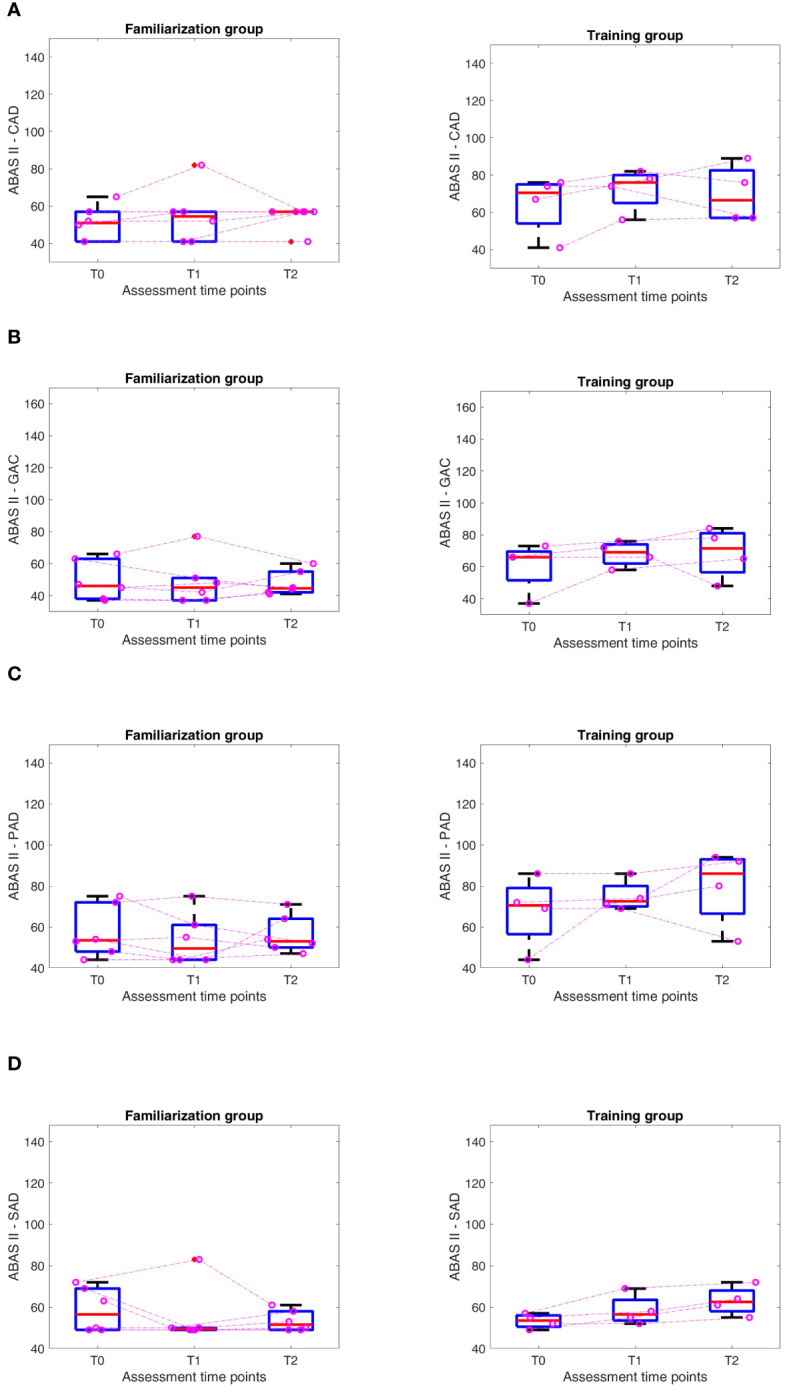
ABAS II Results at different time points (T0-T1-T2) in Familiarization (FAM) and Training (TRAIN) groups for the Conceptual Adaptive Domain (CAD) **(A)**, General Adaptive Composite Score (GAC) **(B)**, Practical Adaptive Domain (PAD) **(C)**, Social Adaptive Domain (SAD) **(D)**. The purple dots represent each of the children, and the dashed lines are an approximation of their evolution.

**Figure 7:**
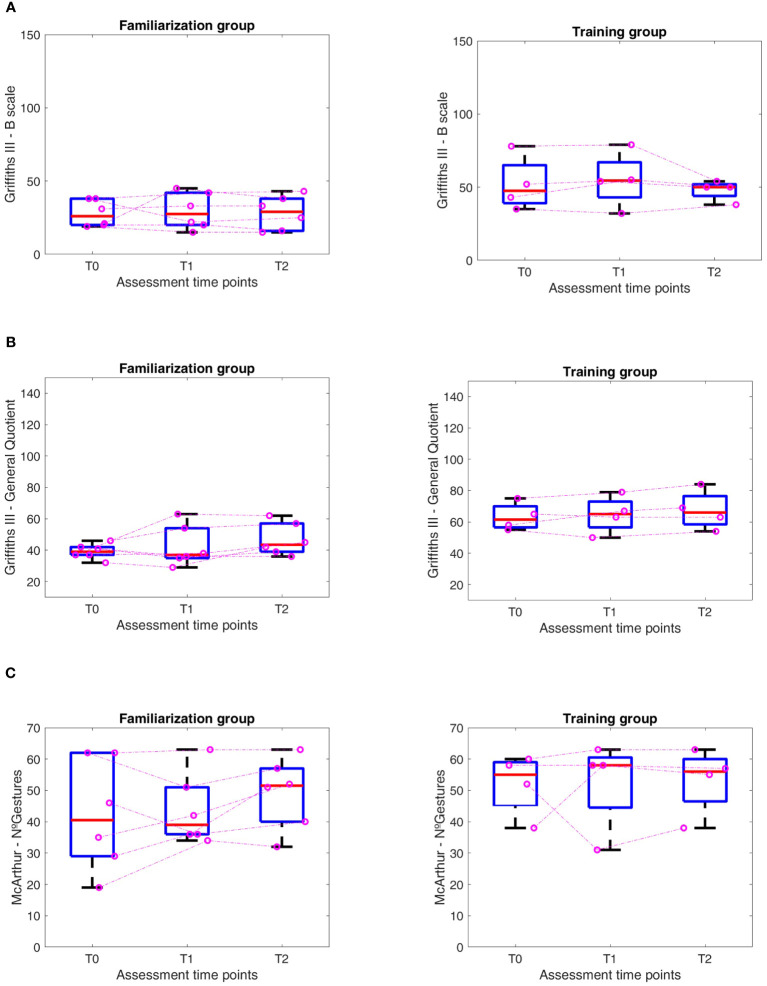
Results at different time points (T0-T1-T2) in Familiarization (FAM) and Training (TRAIN) groups for the **(A)** Griffiths III- B scale, **(B)** Griffiths III – General Quotient, **(C)** McArthur – Number of Gestures.

Analyzing the results for the two groups separately (according to the training level reached) and looking at the medians for the two groups, we found a positive trend in all the scores in the group that reached the training levels. In contrast, in the group that stayed at the familiarization level, we found a trend of improvement only in Griffiths (Scale B) and ABAS-II (CAD) (**Figures 6**, **7**). In the training group, there were significant differences in the Friedman test only for the Social Domain of the ABAS-II questionnaire (p=0.022). For this latter, performing a Wilcoxon test, no differences were found between the several time points.

## 4 Discussion

This pilot study aimed to develop a training protocol to promote intransitive gestures in a triadic interaction (child-robot-operator) in preschoolers with autism spectrum disorder (ASD). We built an experimental protocol based on real-time observation and imitation of the gestures produced by NAO (real-time embodied mirroring). The technological setup allows not only gesture imitation by mirroring the child but also provides automatic feedback on the correct execution of the child through a gesture recognition system (63, 64).

The protocol is based on 14 weeks of weekly sessions of 30 minutes each, divided into five levels; the initial two levels, designated as “familiarization levels,” are designed to facilitate familiarity with NAO and enhance compliance. Levels 3, 4, and 5, designated as “training levels,” comprise training sessions encompassing novel gesture teaching and progressive generalization of the gestures taught within brief narratives and diverse daily life scenarios. At these levels, we have incorporated 17 intransitive communicative gestures commonly utilized in everyday life.

The present study’s first aim was to test the feasibility of the training protocol using the social robot NAO. We found good compliance among the children and families recruited into the study. Despite the problems caused by the COVID-19 pandemic, most children completed the intervention protocol, and none dropped out due to refusal to attend sessions. However, some participants showed fear towards the Robot (due to sensorial stimuli such as lights, sudden movements, or sounds) and needed more familiarization sessions than expected or the presence of a parent in the room during the sessions to be reassured.

Recent studies in literature underlined the potential use of the humanoid robot NAO to encourage interaction in ASD children (23). Increased responses were observed from ASD children to interactions with robots and humans (43). Other studies demonstrated that a protocol therapy using NAO can increase the spontaneous initiative of ASD children in interaction with others (49). Our sample, even if little, showed a non-univocal response to the social robot: one group of children was attracted by the robot and quickly engaged in the interaction; in contrast, another part was initially frightened and needed a more prolonged time of familiarization. We hypothesize that this can be due to the different sensory issues in the children recruited or to their different functional profiles. However, this aspect needs more subjects to understand better how the functional profile at baseline can impact the answer to the social robot.

Regarding the focus on gesture training, our study is in continuity with previous studies (48, 50) that worked on intransitive gesture training. In line with those studies, we decided to use intransitive gestures with a communicative meaning. However, as a point of innovation and in contrast with these works, we did not focus on the learning-action goal itself but on the fostering of triadic interaction: the therapist had an active involvement in the training protocol, and the social robot was used as a facilitator of human-to-human interaction. This aspect is an innovation in this approach, and it is crucial to promote generalization of the skills and promotion of the interaction skills of the child in everyday life. We decided, in fact, not to focus on gesture learning in terms of the exact execution of the motor action. However, we focused on the role of gestures in promoting social communication. As preliminary results, even if we treated a small group of children, we reached some promising results, namely when analyzing separately the groups according to their access to the training levels (levels 3–5). Firstly, we found a trend of improvement for the group that reached the training level, which was maintained even after six months in the follow-up. Secondly, we found a significant difference in the ABAS Social Composite domain in the training group but not in the familiarization group. This is an interesting result, as we found a trend of improvement for the group that worked on gesture training and a significant improvement in adaptive skills related to socialization and play skills in everyday life.

Our study aimed to promote a triadic interaction, using the social robot as a motivator and facilitator while the therapist is meant to be actively involved. This aspect is an innovation in this approach, and it is essential in terms of generalization of the skills and promotion of the child’s interaction skills in everyday life.

As for preliminary results, we found some promising results even if we treated a small group of children.

Focusing on the baseline results, the two groups differ regarding global quotient and adaptive skills but not for autistic symptoms as measured with diagnostic tools. This aspect has been critically analyzed, and we hypothesize that the structure of the training protocol, which focuses on verbal sentences linked to gestures, is more difficult to understand for non-verbal children with severe developmental delay at an earlier age. Our preliminary findings from a small sample suggest that our intervention may be more effective for children with mild to moderate developmental delay. At the same time, we observed a minor engagement in children with more severe developmental delay. This crucial aspect will be further discussed in sections 5 and 6.

Unlike other studies, where children are asked to replicate gestures in a strict imitation protocol (48–50), we included gesture training in a more naturalistic context. This is a controversial point. On the one hand, some authors (48) state that according to the empathizing-systemizing theory (49), a highly structured learning environment leads to positive learning outcomes in children with ASD. A more structured teaching and protocol could be more explicit and promote contingent learning for children with developmental delay and difficulties in receptive communication. On the other hand, the final aim of these studies is not gesture learning itself but its generalization. Therefore, we prioritized engaging children in a more naturalistic setting to promote gesture learning and generalization. Finally, the technological set-up allows not only gesture imitation, providing feedback on the correct execution of the child but also a mirroring of the child and the improvement in accuracy of the robot gesture recognition thanks to the machine learning system used (64).

## 5 Strengths and limitations

This work has many strengths, starting with its focus on ASD, a population that receives substantial scientific attention for the development of new early detection and treatment methods. Our intervention protocol embodies an innovative approach to early intervention.

Moreover, we develop an intervention protocol that involves a humanoid robot, which is another point of great interest in the present scientific landscape, and we insert it in a triadic setting, which means not to substitute the human therapist but aiming to exploit the child’s interest for the robot to foster the human-to-human interaction and promote the acquisition of socio-communicative competences. Compared to the scientific landscape, this novelty needs further work.

About the limitations of the present study, the small sample size and the lack of a control group constituted one of the main drawbacks for the establishment of solid conclusions. Although we conducted a statistical analysis in this group, the related conclusions are influenced by the small sample size, and their impact should be considered accordingly.

Another limitation was the interruption of the intervention due to the Covid-19 pandemic. Even if this disruption after the first two weeks involved few children, it compromised the intended continuity of the protocol, which was meant to be applied over consecutive weeks.

Finally, a limitation of the study is the small number of subjects that reached the training levels. This issue may be due to the difficulty of engaging with the robot using verbal prompts and requests primarily related to intransitive gestures, which can be harder to understand and manage for children with more severe developmental delay. However, children with milder developmental delay participated better in these activities, guiding our critical analysis of the protocol and its future perspectives.

## 6 Implications and future direction

Our pilot study has many implications and powerfully addresses our future work in this field.

Future work will be done by replicating these results, increasing the number of recruited subjects, and including a control group to see if the improvement detected is related to the training proposed. Therefore, the first step will be the design of a Randomized Control Trial study to test the efficacy of this training protocol.

Moreover, adapting the training protocol to engage children with severe developmental delay will be mandatory. A direction could be the implementation of socio-communicative activities. We will add socio-sensorial stimuli, such as tactile, visual, and auditive bids, to encourage children’s exploration and familiarization with the robot, and we will include simple children’s songs, including gestures, to promote children’s involvement and ease familiarization and engagement with the robot.

Furthermore, improvements in the robot technology should be explored through an enlargement of the movement sets, implementation of leg movements (65), and further development of mirroring and gesture recognition algorithms, specifically through machine learning. We are working on identifying and adapting new outcome measures to detect pre- and post-test changes better. Besides the clinical measures, defining quantitative measures to evaluate the rate of shared attention between child and therapist during the triadic interaction can be crucial to assessing our therapy protocol’s impact (49). In addition, as we want to evaluate changes in socio-communicative skills, we are looking for more sensible clinical measures, better at detecting subtle changes, given the brief intervention period and considering the children’s age.

In conclusion, this study tested IOGIOCO, a robotic intervention protocol for children with ASD. This first feasibility study showed some promising results. While current results will need a Randomized Controlled Trial to be confirmed, the present work sets an important milestone in using social robots for ASD treatment, aimed at impacting social and communication skills in everyday life.

## Data availability statement

The datasets presented in this article are not readily available because of ethical reasons. Requests to access the datasets should be directed to sannunziata@dongnocchi.it.

## Ethics statement

The studies involving humans were approved by Ethics Committee of Fondazione Don Gnocchi IRCCS “Santa Maria Nascente”. The studies were conducted in accordance with the local legislation and institutional requirements. Written informed consent for participation in this study was provided by the participants’ legal guardians/next of kin. Written informed consent was obtained from the minor(s)’ legal guardian/next of kin for the publication of any potentially identifiable images or data included in this article.

## Author contributions

SA: Conceptualization, Methodology, Investigation, Data curation, Writing -original draft. LS: Conceptualization, Methodology, Formal analysis, Data curation, Writing – original draft. ArC: Conceptualization, Investigation, Data curation, Writing – original draft. AG: Conceptualization, Methodology, Formal analysis, Writing – review & editing. EB: Conceptualization, Writing – review & editing. EP: Investigation, Writing – review & editing. IO: Conceptualization, Methodology, Investigation, Writing – review & editing. AP: Conceptualization, Methodology, Formal analysis, Writing – review & editing. AnC: Conceptualization, Methodology, Writing – review & editing.
