# Supplementary material for: Interactive mirrOring Games wIth sOCial rObot (IOGIOCO): a pilot study on the use of intransitive gestures in a sample of Italian preschool children with autism spectrum disorder
Source: Front Psychiatry. 2024 Jun 28;15:1356331. doi: 10.3389/fpsyt.2024.1356331 (PMC11240845; doi:10.3389/fpsyt.2024.1356331)
Supplement: Supplementary file 1 [file Table_1.docx]

|  |  |  |  |  |  |  |  |  |  | **GRIFFITHS** | | | | | | **MC ARTHUR** | | | **ABAS II** | | | | | | | | | | | |
| --- | --- | --- | --- | --- | --- | --- | --- | --- | --- | --- | --- | --- | --- | --- | --- | --- | --- | --- | --- | --- | --- | --- | --- | --- | --- | --- | --- | --- | --- | --- |
|  |  |  |  |  |  |  |  |  |  | **B SCALE** | | | **GQ** | | | **n° GEST** | | | **GAC** | | | **CAD** | | | **SAD** | | | **PAD** | | |
|  | **I.D.** | **ADI**  **SI** | **ADI**  **CL** | **ADI**  **RRP** | **ADOS**  **SA** | **ADOS**  **RRP** | **ADOS**  **CS** | **Training**  **Level** | **Age** | **T0** | **T1** | **T2** | **T0** | **T1** | **T2** | **T0** | **T1** | **T2** | **T0** | **T1** | **T2** | **T0** | **T1** | **T2** | **T0** | **T1** | **T2** | **T0** | **T1** | **T2** |
| **Training** | **9** | 1,53 | 1,39 | 0,83 | 1,1 | 1,5 | 7 | 5 | 62 | 52 | 54 | 50 | 65 | 63 | 63 | 60 | 63 | 63 | 66 | 66 | 48 | 74 | 74 | 57 | 52 | 52 | 55 | 69 | 69 | 53 |
|  | **8** | 1,4 | 1,5 | 1,3 | 1,8 | 1,25 | 9 | 5 | 67 | 43 | 55 | 50 | 58 | 67 | 69 | 58 | 58 | 57 | 73 | 76 | 78 | 76 | 82 | 76 | 55 | 58 | 64 | 86 | 86 | 92 |
|  | **6** | 1,4 | 1,5 | 1,3 | 0,8 | 1 | 6 | 5 | 50 | 78 | 79 | 54 | 75 | 79 | 84 | 52 | 31 | 38 | 66 | 72 | 84 | 67 | 78 | 89 | 57 | 69 | 72 | 72 | 74 | 94 |
|  | **5** | 1,85 | 1,86 | 0,5 | 2 | 1,25 | 9 | 3 | 63 | 35 | 32 | 38 | 55 | 50 | 54 | 38 | 58 | 55 | 37 | 58 | 65 | 41 | 56 | 57 | 49 | 55 | 61 | 44 | 71 | 80 |
| **Familiarisation** | **1** | 1 | 0,8 | 1,5 | 1,6 | 1,5 | 7 | 2 | 55 | 38 | 42 | 43 | 46 | 54 | 57 | 62 | 63 | 63 | 66 | 77 | 60 | 65 | 82 | 57 | 72 | 83 | 61 | 72 | 75 | 71 |
|  | **4** | 1,4 | 1,86 | 1 | 1 | 0,75 | 6 | 2 | 68 | 31 | 33 | 33 | 37 | 38 | 45 | 29 | 36 | 51 | 45 | 48 | 45 | 57 | 57 | 57 | 50 | 50 | 53 | 53 | 55 | 50 |
|  | **7** | 1,87 | 1,7 | 0,8 | 1,1 | 1,75 | 6 | 1 | 55 | 20 | 20 | 16 | 32 | 29 | 42 | 19 | 34 | 32 | 37 | 37 | 42 | 41 | 41 | 57 | 49 | 49 | 49 | 44 | 44 | 47 |
|  | **2** | 1,4 | 1,75 | 1,5 | 1 | 1,5 | 6 | 1 | 56 | 21 | 45 | 38 | 37 | 63 | 62 | 46 | 36 | 40 | 47 | 42 | 55 | 50 | 57 | 57 | 63 | 50 | 58 | 54 | 44 | 64 |
|  | **3** | 1,54 | 0,6 | 0,67 | 1,5 | 0,75 | 6 | 2 | 63 | 19 | 15 | 15 | 41 | 36 | 36 | 62 | 51 | 57 | 63 | 51 | 44 | 52 | 52 | 57 | 69 | 49 | 50 | 75 | 61 | 52 |
|  | **11** | 1,5 | 1,75 | 0,5 | 1 | 1 | 5 | 1 | 50 | 38 | 22 | 25 | 42 | 35 | 39 | 35 | 42 | 52 | 38 | 37 | 41 | 41 | 41 | 41 | 49 | 49 | 49 | 48 | 44 | 54 |

Supplementary table 1. Comprehensive clinical results, divided for rehabilitation level reached.

Legend: ADI-R Social interaction (SI); Communication and language (CL); Restricted and repetitive behaviours (RRB), ADOS module (mod); ADOS Social Affect (SA); ADOS Restrictive Repetitive Behaviours (RRB) ADOS Comparative Score (CS); Griffiths-III General Quotient (GQ); General Adaptive Composite Score (GAC), Conceptual Adaptive Domain (CAD); Social Adaptive Domain (SAD); Practical Adaptive Domain (PAD)
